# The relationship of systemic inflammation to prior hospitalization in adult patients with cystic fibrosis

**DOI:** 10.1186/1471-2466-12-3

**Published:** 2012-02-14

**Authors:** David A Ngan, Pearce G Wilcox, May Aldaabil, Yuexin Li, Jonathon A Leipsic, Don D Sin, SF Paul Man

**Affiliations:** 1 UBC James Hogg Research Centre, Institute for Heart + Lung Health at St. Paul's Hospital and Department of Medicine, University of British Columbia, Vancouver, BC, Canada

## Abstract

**Background:**

In cystic fibrosis (CF) patients, it has been suggested that systemic inflammation may be an important risk factor for poor health outcomes. The relationship of plasma inflammatory biomarkers to lung function and hospitalization history remains largely unexplored.

**Methods:**

This cross-sectional study included 58 consecutive, clinically stable adults from the CF Clinic at St. Paul's Hospital (Vancouver, Canada). Blood levels of interleukin (IL)-6, IL-1β, C-reactive protein (CRP), interleukin (IL)-6, IL-1β, granzyme B (GzmB), chemokine C-C motif ligand 18 (CCL18/PARC), surfactant protein D (SP-D), lipopolysaccharide (LPS)-binding protein, and soluble cluster of differentiation 14 (sCD14) were measured using enzyme-linked immunosorbent assays, and LPS levels were measured using a *Limulus *amebocyte lysate assay. Spirometry was also performed. Multivariable linear regression analysis was used to assess relationships of the blood biomarkers to lung function.

**Results:**

Lung function impairment was independently associated with elevated plasma levels of CRP (*P *< 0.01), IL-6 (*P *= 0.04), IL-1β (*P *< 0.01), and LBP (*P *< 0.01). Increasing age (*P *< 0.01), reduced body mass index (*P *= 0.02), prior hospitalizations (*P *= 0.03), and presence of *Pseudomonas aeruginosa *in sputum cultures (*P *< 0.01) were also associated with reduced lung function. Elevated concentrations of LPS in plasma were associated with a previous history of hospitalization (*P *< 0.05). There was a trend towards an increase in plasma IL-6 (*P *= 0.07) and IL-1β (*P *= 0.06) levels in patients who were previously hospitalized.

**Conclusions:**

IL-6 and IL-1β are promising systemic biomarkers for lung function impairment and history of hospitalization in adult patients with CF.

## Background

Cystic fibrosis (CF) is a progressive, debilitating disease that affects nearly 30,000 Americans and occurs with a frequency of about 1 in 3500 births [1]. It is characterized by persistent lung infection and lung function impairment. It also affects other organs including the sinuses, gastrointestinal tract, endocrine glands, and the bone [2-5]. Although all CF cases are caused by a mutation in the gene for the CF transmembrane conductance regulator, there is considerable heterogeneity in the rate at which the disease progresses [6]. The traditional risk factors for rapid progression include reduced body mass index, colonization of the airways with pathogenic bacteria such as *Pseudomonas aeruginosa*, and female sex [7-9]. More recently, some have suggested that systemic inflammation may be another important risk factor for poor health outcomes in CF independent of these traditional risk factors [3,8,10,11]. However, the studies that have evaluated this issue have produced inconsistent results and have measured different components of the immune system, making cross comparisons difficult. Moreover, none of these studies have evaluated these biomarkers on hard clinical outcomes such as exacerbations or hospitalizations, which are important endpoints in CF. In this study, we determined the relationship of plasma inflammatory biomarkers to lung function and hospitalization history in adult patients with CF. The plasma biomarkers were carefully chosen to represent innate or adaptive immunity, a by-product of Gram-negative pathogens, or lung-based proteins.

## Methods

### Study Population and Blood Collection

We enrolled 58 consecutive adult patients from the Cystic Fibrosis (CF) Clinic at St. Paul's Hospital (Vancouver, British Columbia, Canada) between April and December 2009, who were clinically stable at the time of assessment. For inclusion, patients had to have one or more clinical features consistent with the CF phenotype [12] as well as a genotype with two identifiable disease-causing CF transmembrane conductance regulator (CFTR) mutations and sweat chloride measurements greater than 60 mmol/L on two occasions. Patients who had an exacerbation within the previous 4 weeks were excluded from the study. This study was conducted with the approval of the University of British Columbia - Providence Health Care Research Ethics Board (UBC-PHC REB). Following informed consent, we collected venous blood samples and performed spirometry using standard techniques, in accordance with guidelines from the American Thoracic Society [13]. Demographic and clinical data were obtained by chart review.

### Clinical Information

The subjects' infection status was determined by microbial review within the preceding 3 years of study entry. Those who had at least one sputum culture positive of *Pseudomonas aeruginosa *were considered to be infected by this organism. We also performed chart review and retrieved data from the hospital database to determine whether the patients had a hospitalization for CF exacerbation in the previous 5 years.

### Biomarker Assays

Plasma was prepared from the collected blood samples and a select number of circulating inflammatory proteins were measured using high-sensitivity enzyme-linked immunosorbent assay (ELISA) kits that were commercially available. These included interleukin (IL)-6 and IL-1β (R&D Systems, Minneapolis, MN), cytokines involved in the early phase inflammatory response; C-reactive protein (CRP; R&D Systems), an acute phase response protein; granzyme B (GzmB; eBioscience, San Diego, CA), a protein involved in adaptive immunity; chemokine C-C motif ligand 18/pulmonary and activation-regulated chemokine (CCL18/PARC; R&D Systems) and surfactant protein D (SP-D; Biovendor, Brno, Czech Republic), pneumo-proteins (i.e. proteins synthesized predominantly in the lungs) [14,15]; and LPS-binding protein (LBP; Hycult Biotech, Uden, The Netherlands) and soluble cluster of differentiation 14 (sCD14; R&D Systems), proteins involved in lipopolysaccharide (LPS) signalling. The coefficients of variation for these kits were 7.4%, 12.3%, 3.1%, 5.8%, 1.2%, 2.2%, 1.7%, and 5.9% respectively, and the lower detection limits were 0.039 pg/mL, 0.057 pg/mL, 0.010 ng/mL, 0.2 pg/mL, 0.01 ng/mL, and 0.2 ng/mL, 4.4 ng/mL, and 0.125 ng/mL respectively. LPS levels were measured using a commercially-available kinetic chromogenic *Limulus *amebocyte lysate (LAL) assay kit (Lonza Walkersville, Walkersville, MD) following plasma dilution and heat inactivation pre-treatment steps to diminish interference from plasma proteins. The coefficient of variation for the assay was 2.2%, and the lower detection limit was 0.5 pg/mL. Of these biomarker assays, only the IL-1β ELISA had samples with values below the limit of detection (18 undetectable out of 58 samples, or 31%). In plasma samples, the manufacturers' reported ranges for IL-6, IL-1β, CRP, GzmB, and sCD14 measurements in healthy volunteers were 0.428 to 8.8 pg/mL, non-detectable to 0.452 pg/mL, 104 to 4185 ng/mL, 0.8 to 24.1 pg/mL, and 1200 to 3100 ng/mL, respectively. Reference ranges were not available for CCL18/PARC, SP-D, LBP, and LPS, and the dynamic ranges of these assays were 7.8 to 500 pg/mL, 1.56 to 100 ng/mL, 4.4 to 50 ng/mL, and 0.5 to 500 pg/mL, respectively.

### Statistical Analysis

Data for the biomarker measurements were analyzed after natural log transformation due to their skewed distribution, and values below the limit of detection were assigned the value of the lower detection limit for the particular kit. The relationships of the biomarkers to lung function (FEV_1 _as a percentage of predicted) and to each other were assessed using linear regression. Multivariable linear regression analysis was performed to assess the role of possible confounding factors such as age, sex, and pseudomonal status. Plasma biomarker levels were compared between those who did and did not experience a hospitalization in the past using a Student's *t*-test for independent samples. Fisher's exact test was used to analyze categorical data. The independent relationship of plasma biomarkers to FEV_1% _predicted was ascertained using multiple regression analysis. To ensure parsimony and enhance the robustness of the model, we used a stepwise approach to select only those covariates that significantly impacted on the relationship (the significance level for entering, *P *≤ 0.05 and the significance level for stay, *P *≤ 0.05). To facilitate interpretation and cross-comparisons of beta-coefficients of plasma biomarkers from the regression model, we standardized the beta-coefficients to their standard deviation. Thus, the beta-coefficients are presented per 1 SD increase in the plasma biomarker expression. *P*-values less than 0.05 were considered significant (two-tailed test). All analyses were conducted using SAS (Carey, N.C.) version 9.1.

## Results

### Patient Demographics

The age of the study subjects ranged from 18 to 61 years (Table 1). The baseline clinical characteristics of the study subjects are summarized in Table 1 in two groups based on the median value of FEV_1% _predicted. Greater mean age, reduced BMI, hospitalizations in the previous 5 years, and presence of *Pseudomonas aeruginosa *in sputum cultures were all significantly associated with below-median FEV_1% _predicted values. Of the 58 subjects, 21 had been hospitalized (36% of total) and 37 had not. 42 of the subjects were classified as *Pseudomonas*+ based on sputum microbiology (72.41% of total) and 16 were *Pseudomonas*-. A greater portion of patients with below-median FEV_1% _predicted values had been administered Azithromycin and Tobramycin versus patients with above-median FEV_1% _predicted values.

**Table 1:** Patient demographics and clinical characteristics

	Total (n = 58)	FEV_1% _predicted ≥ median (n = 29)	FEV_1% _predicted < median (n = 29)	*P*-value
Age, years range	18 - 61	18 - 49	19 - 61	< 0.01
Sex, male n (%)	34 (58.62)	18 (62.1)	16 (55.2)	0.79
BMI, kg/m^2 ^± SD	23.24 ± 3.25	24.2 ± 3.4	22.3 ± 2.9	0.02
FEV_1_, % predicted ± SD	71.93 ± 24.80	92.9 ± 12.9	51.0 ± 13.2	-
Current medications				
Azithromycin, n (%)	18 (31.03)	3 (10.3)	15 (51.7)	< 0.01
Ciprofloxacin, n (%)	2 (3.45)	1 (3.4)	1 (3.4)	1.00
Dornase alfa, n (%)	24 (41.38)	11 (37.9)	13 (44.8)	0.79
Ibuprofen, n (%)	2 (3.45)	2 (6.9)	0 (0)	0.49
Prednisone, n (%)	0 (0)	0 (0)	0 (0)	-
Tobramycin, n (%)	13 (22.41)	2 (6.9)	11 (37.9)	0.01
Inhaled steroids, n (%)	37 (63.79)	15 (51.7)	22 (75.9)	0.10
Diabetes, n (%)	22 (37.93)	10 (34.5)	12 (41.4)	0.79
Hospitalization, n (%)	21 (36.2)	6 (20.7)	15 (51.7)	0.03
*Pseudomonas*, n (%)	42 (72.41)	15 (51.7)	27 (93.1)	< 0.01

### Systemic Inflammation and Lung Function

**Figure 1:**
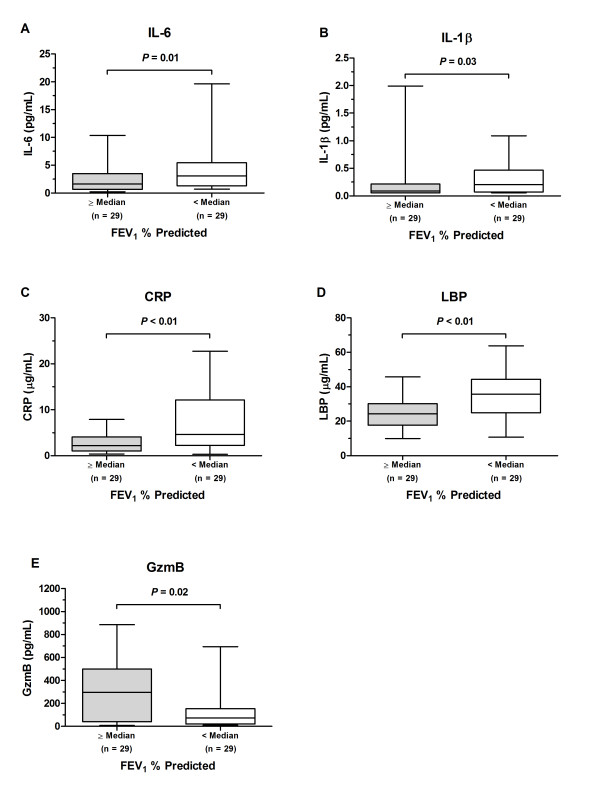
**Plasma biomarker concentrations in cystic fibrosis subjects with above-median (n = 29) and below-median (n = 29) FEV_1% _predicted values**. (A-D) Geometric mean plasma levels of IL-6, IL-1β, CRP, and LBP were higher in subjects with below-median FEV_1% _predicted values than in those with above-median FEV_1% _predicted values. (E) The geometric mean plasma level of GzmB was lower in subjects with below-median FEV_1% _predicted values than in those with above-median FEV_1% _predicted values.

CRP, IL-6, IL-1β, and LBP were significantly correlated with lung function impairment in both univariable and multivariable analysis (Table 2). LPS was significantly related to FEV_1 _only in the multivariable analysis. The use of standardized beta-coefficient (i.e. the change in FEV_1_% predicted per 1 standard deviation increase in the plasma concentrations of the biomarker) allows for comparison of the beta-coefficients across biomarkers. CRP had the highest standardized beta-coefficient, followed by LBP, IL-6 and IL-1β, suggesting that CRP is most strongly associated with FEV_1_. Geometric mean plasma levels of IL-6, IL-1β, CRP, and LBP of patients with below-median FEV_1% _predicted values were 3.0 pg/mL, 0.21 pg/mL, 4.6 μg/mL, and 33.2 μg/mL, respectively. These were all significantly higher than in patients with above-median FEV_1% _predicted values (1.6 pg/mL, 0.12 pg/mL, 2.0 μg/mL, and 22.9 μg/mL, respectively) (Figure 1). The geometric mean plasma level of GzmB was significantly lower in patients with below-median FEV_1% _predicted values (71.6 pg/mL) compared to those with above-median FEV_1% _predicted values (166.3 pg/mL).

**Table 2:** Relationship between FEV_1 _percent predicted and biomarkers in CF subjects per 1 log increase in the biomarkers (n = 58)

	Unadjusted		Adjusted*		Standardized Beta-Coefficient (per 1 SD increase in the levels of biomarker)
**Biomarker**	**β ± SE**	***P*-value**	**β ± SE**	***P*-value**	

CRP, mg/L	-10.61 ± 2.65	< 0.01	-7.04 ± 1.98	< 0.01	-0.3134
IL-6, pg/mL	-10.72 ± 3.11	< 0.01	-5.53 ± 2.66	0.04	-0.2159
IL-1β, pg/mL	-6.97 ± 3.36	0.04	-4.65 ± 2.67	< 0.01	-0.1784
SP-D, μg/mL	-17.70 ± 7.95	0.03	-9.83 ± 5.93	0.10	-0.1584
CCL18, pg/mL	-6.33 ± 5.93	0.29	-3.01 ± 4.23	0.48	-0.0672
GzmB, pg/mL	4.09 ± 2.27	0.08	-0.33 ± 1.89	0.86	-0.0187
LPS, pg/mL	1.07 ± 7.44	0.89	13.30 ± 5.41	0.02	0.0239
sCD14, μg/mL	1.88 ± 9.67	0.85	-10.26 ± 6.96	0.15	-0.1414
LBP, μg/mL	-22.28 ± 6.63	< 0.01	-16.02 ± 4.74	< 0.01	-0.2946

* Adjusted for age, pseudomonal status, and history of hospitalization, which were significantly related to reduced lung function

### Circulating Systemic Inflammatory Biomarkers and Hospitalization History

**Figure 2:**
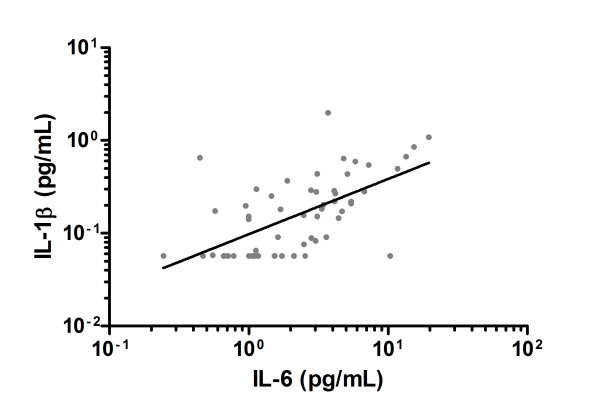
**Plasma IL-6 was significantly correlated with plasma IL-1β in cystic fibrosis subjects (n = 58)**. β ± SE = 0.595 ± 0.105; IL-6 and IL-1β ln-transformed. R^2 ^= 0.37; *P *< 0.01.

The geometric mean plasma levels of IL-6, IL-1β, and LPS of hospitalized CF subjects were 3.6 pg/mL, 0.19 pg/mL, and 1.3 ng/mL, respectively. These were all significantly higher than in non-hospitalized subjects (1.7 pg/mL, 0.10 pg/mL, and 0.97 ng/mL, respectively) (Table 3). After adjustment for FEV_1% _predicted, BMI and pseudomonal status in sputum, the relationships weakened (Table 3). There was a significant association between plasma IL-6 levels and IL-1β levels (Figure 2). Geometric mean plasma concentrations of CRP, GzmB, CCL18, SP-D, LBP, and sCD14 did not significantly differ between the previously hospitalized and non-hospitalized groups (Table 3). The c-statistic of FEV_1_, BMI and pseudomonal status together was 0.797. There was a modest improvement in the c-statistic by adding IL-6, IL-1β, or LPS (0.837, 0.828, and 0.841, respectively).

**Table 3:** Geometric means (and interquartile ranges) of biomarkers in CF patients who were and were not previously hospitalized

Biomarker	Total (n = 58)*	Previously hospitalized (n = 21)*	Not previously hospitalized (n = 37)*	*P*-value	**Adjusted *P*-value**^**†**^	c-statistics
CRP, μg/mL	3.0 (1.5, 6.1)	3.5 (2.0, 8.0)	2.8 (1.3, 5.6)	0.50	0.51	0.795
						
IL-6, pg/mL	2.2 (1.1, 4.2)	3.6 (2.8, 5.8)	1.7 (1.0, 3.1)	< 0.01	0.07	0.837
						
IL-1β, pg/mL	0.16 (0.06, 0.29)	0.19 (0.06, 0.50)	0.10 (0.06, 0.20)	< 0.01	0.06	0.828
						
SP-D, ng/mL	84.3 (64.0, 112.3)	87.5 (75.3, 106.1)	82.6 (63.7, 113.2)	0.60	0.26	0.804
						
CCL18, ng/mL	59.5 (41.4, 75.3)	60.1 (38.7, 73.5)	59.2 (44.5, 75.9)	0.92	0.92	0.797
						
GzmB, pg/ml	109.2 (25.5, 389.8)	90.7 (25.4, 384.9)	121.2 (26.2, 389.8)	0.46	0.83	0.792
						
LPS, ng/mL	1.1 (0.9, 1.3)	1.3 (1.0, 1.4)	1.0 (0.8, 1.3)	0.01	0.04	0.841
						
sCD14, μg/mL	1.1 (0.9, 1.3)	1.1 (1.0, 1.3)	1.1 (0.8, 1.3)	0.89	0.49	0.789
						
LBP, μg/mL	27.6 (22.0, 39.9)	30.8 (23.7, 41.2)	25.9 (18.8, 35.7)	0.17	0.79	0.781

* Data are presented as geometric mean (25^th^, 75^th ^percentile)

^† ^Adjusted for FEV_1% _predicted, BMI, and pseudomonal status, which collectively had a c-statistic (or area under the curve) value of 0.797

## Discussion

This was a unique study investigating the relationship of systemic inflammation to lung function impairment and hospitalization history among clinically stable CF patients. This study produced several important findings. First, biomarkers that are related to innate immunity or early acute phase reactants such as IL-6, IL-1β, CRP, and LBP were significantly associated with reduced lung function in CF patients independent of age, pseudomonal status, or history of hospitalization, suggesting that systemic inflammation is an independent risk factor for disease progression in CF. The directionality of the relationship is uncertain. Thus, it remains unknown whether the rise in these biomarkers is the result or the cause of impaired lung function. Our data extend the findings of a previous study that examined a cohort of adult CF patients aged 30 years or greater. Levy *et al. *found an association between lower FEV_1 _percent predicted and higher serum CRP levels, but they did not adjust for sex or pseudomonal status of the patients, and the study cohort was limited to an older population [11]. Furthermore, their retrospective study design prevented them from obtaining serum samples and performing pulmonary function tests within a close proximity of time. While our current study did not make comparisons to healthy controls, previous studies have found that median plasma or serum concentrations of IL-6, IL-1β, IL-1 receptor antagonist (IRAP) [16], neutrophil granule proteins, and CRP [17] were higher in CF patients versus healthy subjects. Collectively, the use of plasma markers of systemic inflammation, especially IL-6 and CRP, provides additional indicators of clinical status and may add to our understanding of the relationship between inflammation and the severity of lung disease in CF patients.

Second, in addition to the traditional factors such as reduced BMI, poor lung function as measured by FEV_1 _percent predicted, and presence of *Pseudomonas aeruginosa *in sputum cultures, we found that plasma levels of two early phase inflammatory cytokines, IL-6 and IL-1β, were significantly associated with prior hospitalization in patients with CF, independent of the traditional factors. However, there was no significant relationship of GzmB, a marker of adaptive immunity, lung-based proteins such as CCL18/PARC and SP-D, or acute-phase reactants such as CRP, LBP, and sCD14 [18] with hospitalization history. Together, these data suggest that early phase inflammatory cytokines may be good candidate plasma biomarkers of health outcomes in CF.

Our third important finding was that plasma LPS derived from Gram-negative bacteria is significantly higher in those who were previously hospitalized for a CF exacerbation than those who were not. LPS is an immunologically active antigen, which can cause an intense inflammatory process in the lung and elsewhere. Its presence in the systemic circulation may enhance the systemic inflammatory response in CF, as previously seen in a murine model [19]. We postulate that some of the LPS expression in the systemic circulation may be derived from the lungs through a process called translocation. It is conceivable that the diseased respiratory tract in CF may facilitate translocation of bacterial components or pro-inflammatory cytokines from the lungs to the systemic circulation where it incites an inflammatory response. This biological plausibility is supported by a study in rabbits where it was shown that it is physically possible for LPS to undergo pulmonary-to-systemic translocation under certain conditions, specifically in mechanical ventilation strategies [20]. While our methods did not allow us to determine the originating source of plasma LPS, we speculate that the LPS we measured is likely derived from *P. aeruginosa *in the lungs or other Gram-negative, CF-related bacteria. Future studies will be needed to test this hypothesis.

There were important limitations to our study. This is a cross-sectional study, which precludes firm conclusions on causality or directionality of the relationship. While we postulate that systemic inflammation drives disease progression, it is entirely possible that disease progression is responsible for systemic inflammation, and a comprehensive prospective longitudinal study would be needed to address this issue. Longitudinal data may also provide insight into plasma biomarker profiles during acute exacerbations and following antibiotic treatment. Additionally, we did not measure other pro-inflammatory biomarkers such as TNF-α and IL-8 or those with known anti-inflammatory effects such as IL-10, which has been shown in a mouse model to reduce the inflammatory response to *Pseudomonas aeruginosa *[21]. Future investigation into these regulators of the inflammatory response could provide a clearer picture of the complex interactions involved that lead from excessive inflammation to disease progression.

## Conclusions

Notwithstanding the limitations, we found that more intense systemic inflammation, mediated by the innate immune system, is associated with prior CF hospitalizations and with lung function impairment in CF. IL-6 and IL-1β, in particular, are promising systemic biomarkers for disease progression and hospitalization in CF. A large prospective study testing these promising biomarkers would be of great value in determining their usefulness as a clinical tool in managing patients with CF.

## Competing interests

The authors declare that they have no competing interests.

## Authors' contributions

DAN carried out the immunoassays, contributed to the acquisition and interpretation of the data, performed statistical analysis, and drafted the manuscript for important intellectual content; PGW participated in the design of the study and contributed to the acquisition and interpretation of the data; MA contributed to the acquisition and interpretation of the data; YL contributed to the acquisition of the data; JAL participated in the design of the study and contributed to the acquisition and interpretation of the data; DDS participated in the design of the study, performed statistical analysis, contributed to interpretation of the data, and drafted the manuscript for important intellectual content; SFPM conceived the study, participated in the design of the study, contributed to interpretation of the data, and drafted the manuscript for important intellectual content. All authors read and approved the final manuscript.

## Pre-publication history

The pre-publication history for this paper can be accessed here:

http://www.biomedcentral.com/1471-2466/12/3/prepub
